# Material and Optical Properties of Fluorescent Carbon Quantum Dots Fabricated from Lemon Juice via Hydrothermal Reaction

**DOI:** 10.1186/s11671-018-2581-7

**Published:** 2018-06-07

**Authors:** Meiqin He, Jin Zhang, Hai Wang, Yanrong Kong, Yiming Xiao, Wen Xu

**Affiliations:** 1 School of Physics and Astronomy, Yunnan University, Kunming, 650091 People’s Republic of China; 2 Key Laboratory of Yunnan Provincial Higher Education Institutions for Organic Optoelectronic Materials and Device, Kunming University, Kunming, 650214 People’s Republic of China; 3 Institute of Solid State Physics, Chinese Academy of Sciences, Hefei, 230031 People’s Republic of China

**Keywords:** Carbon quantum dot, Photoluminescence, Lemon juice, Cell imaging

## Abstract

The water-soluble fluorescent carbon quantum dots (CQDs) are synthesized by utilizing lemon juice as carbon resource via a simple hydrothermal reaction. The obtained CQDs are with an average size of 3.1 nm. They reveal uniform morphology and well-crystalline and can generate bright blue-green light emission under UV or blue light irradiation. We find that the fluorescence from these CQDs is mainly induced by the presence of oxygen-containing groups on the surface and edge of the CQDs. Moreover, we demonstrate that the as-prepared CQDs can be applied to imaging plant cells. This study is related to the fabrication, investigation, and application of newly developed carbon nanostructures.

## Background

Carbon quantum dot (CQD) is a new class of carbon-based nanomaterial normally with the spatial size less than 20 nm, which was discovered by Xu et al. in 2004 [1]. The fluorescent carbon nanoparticles were fabricated by Sun et al. via laser ablation of graphite powder in 2006 [2] and have been named as “carbon quantum dots (CQDs)” since then. The fluorescent CQDs have a great potential to be applied in photocatalysis, optoelectronic devices, biomedicine, thin film display, healthy lighting, and other disciplines of practical applications. Compared with traditional semiconductor-based quantum dots, the CQDs can be obtained by low-cost fabrication techniques and have fascinating and important features such as good bio-compatibility, precise biological target, low toxicity, and stronger quantum size effect. In recent years, the fluorescent CQDs have attracted a tremendous attention [3, 4] due to their excellent structural and optical properties [5]. They have been proposed as substitution materials for conventional semiconductor quantum dots in the application areas including biological imaging, biological labeling, quantum dot LED (QLED), environmental protection, and other related fields [6–9]. The research on CQDs has been growing fast in condensed matter physics, material science, electronics, and optoelectronics. Related fundamental and application studies have been extensively undertaken around the world [3–9].

At present, there are diverse techniques [10, 11] to synthesize CQDs, such as hydrothermal approach [11, 12], microwave method [13], and so on. The CQDs have been synthesized from various carbon precursors such as glucose [14], citric acid [15], and ascorbic acid [16]. However, the technique for efficient fabrications of biocompatible fluorescent CQDs on a large production scale is still in need and has become a challenge for practical applications of the CQDs. It has been noticed that the direct synthesis of the CQDs from food products [17–19] and/or by-products [20] is one of the promising and significant strategies. Red-emitting carbon dots (R-CDs) with an average diameter of 4 nm and a high quantum yield (QY) of 28% in water were synthesized [21] by heating an ethanol solution of pulp-free lemon juice. A strong reductant NaBH_4_ added into the R-CDs was used as a means of increasing the intensity of light emission from the R-CDs. However, we know that NaBH_4_ is toxic. Very recently, we have fabricated the green- and blue-emitting CQDs from tofu wastewater without adding any toxic substances [22]. The CQDs made from food products and/or by-products are considered be safe for biological applications because there is almost no known toxicity in these natural carbon resources. Recently, several serious investigations have been carried out to synthesis CQDs from non-toxic carbon resources using one-step approach and a significant progress has been achieved in the synthesis, study, and application of these CQDs. For example, garlic was used as a green source to synthesize CQDs [23]. Detailed structural and composition studies demonstrated [23] that the content of N and the formation of C–N and C=N are keys to improve the photoluminescence (PL) QY. Furthermore, the CQDs exhibit excellent stability in a wide pH range and high NaCl concentrations, rendering them applicable in complicated and harsh conditions [23].

The prime motivation of the present work is to develop a simple and efficient experimental method for low-cost fabrication of CQDs from lemon juice by using hydrothermal treatment at relatively low temperatures and through a less time-consuming process. It is known that lemon juice can be easily and cheaply obtained, and therefore, it is a good source of carbon for CQD-based sample and device fabrication. Compared with the previous study [21], the non-toxic CQDs obtained in our work are more suitable for biological imaging and cell markers. In this study, we also conduct the examination of the basic material and optical properties of the CQDs realized from lemon juice and apply the CQDs to imaging plant cells.

## Methods

### Precursory Materials

In this study, the carbon precursory materials are taken from fresh lemon juice. The major ingredients and their percentages are obtained by high-performance liquid chromatography (HPLC) measurement as shown in Table 1. For sample preparation, the fresh lemon taken as a carbon source and fresh onion used for cell imaging were purchased from the local supermarket. The ethanol was analytically pure and used as dispersing agent. Deionized water (18.25 MΩ cm) was used for the experiments.

**Table 1:** Major ingredients of fresh lemon juice

Ingredients	Content (%)
Citric acid	6.30
Total sugar	0.93
Carbohydrate	0.93
Protein	0.38
Cellulose	0.10
Vitamin C	0.02
Vitamina B1, B2, fat, etc.	0.34
Water	91.00

### Synthesis of CQDs

**Fig. 1:**
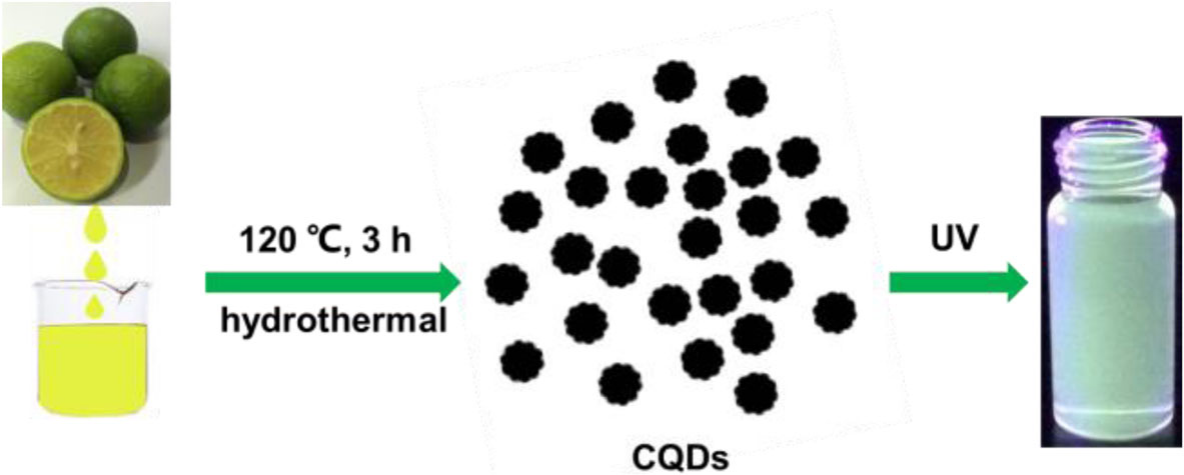
Preparation of CQDs from lemon juice by hydrothermal treatment

The CQDs were synthesized from lemon juice by a simple hydrothermal treatment at relatively low temperatures and through a less time-consuming process. The typical sample preparation processes are shown in Fig. 1. Eighty milliliters of pulp-free lemon juice was mixed with 60 mL of ethanol. The mixture was then transferred into a polytetrafluoroethylene-equipped stainless steel autoclave and is heated at a constant temperature at about 120 °C for 3 h. After the reaction, the dark brown product was obtained after natural cooling to room temperature. The dark brown solution was washed with excess dichloromethane to remove the unreacted organic moieties and this step can be repeated 2–3 times. The deionized water was added until the volume of the brown solution increased up to one third of the solution and centrifuged at 10000 rpm for 15 min to separate the large particles. Thus, the CQD samples can be obtained by carbonization of lemon juice, which contains carbohydrates and organic acids like glucose, fructose, sucrose, ascorbic acid, citric acid, etc. as carbon precursors. Our facile hydrothermal reaction is at a lower temperature (120 °C) and takes less time (3 h), compared to the reported method [24].

### Characterization

The morphology and microstructures of the CQDs realized from lemon juice were analyzed by the transmission electron microscope (JEM 2100, Japan) operated at 300 KV. The crystalline phase of the CQDs was investigated by X-ray diffraction (Rigaku TTR-III, Japan) using Cu-Kα radiation (*λ* = 0.15418 nm). The UV-Vis absorption spectrum was measured by a UV-Vis spectrophotometer (Specord200). The photon-induced light emission was examined by fluorescence spectrophotometer (IHR320, HORIBA Jobin Yvon, USA) for different excitation wavelengths ranging from 330 to 490 nm. The X-ray photoelectron spectroscopy (XPS) spectra were recorded by PHI5000 Versa Probe II photoelectron spectrometer with Al Kα at 1486.6 eV.

## Results and Discussions

**Fig. 2:**
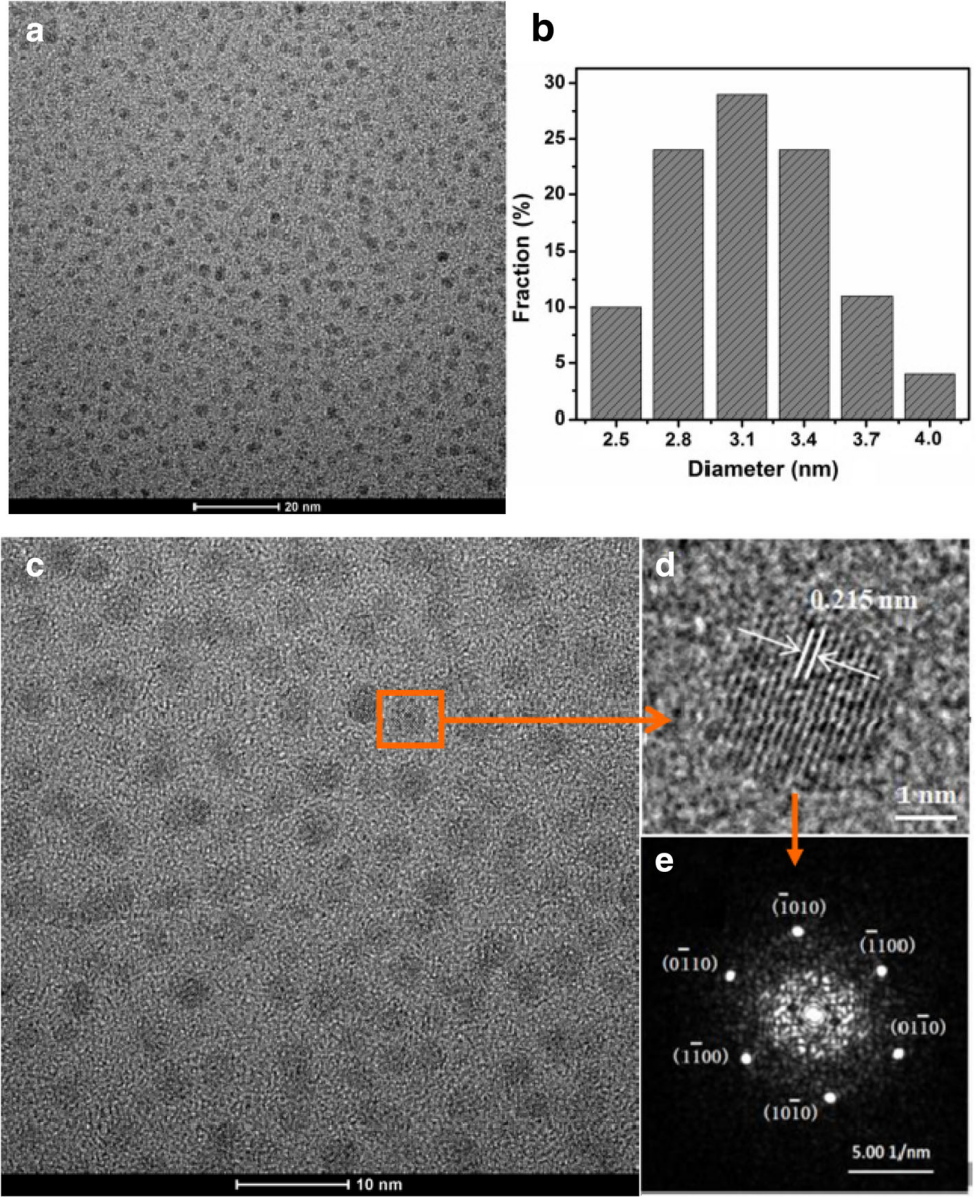
**a**, **c**, **d** TEM image. **b** Particle size distribution of CQDs. **e** The corresponding FFT pattern of CQDs

The transmission electron microscope (TEM) images of CQDs are shown in Fig. 2. The low magnification TEM image of the as-prepared samples indicates that CQDs have a uniform dispersity. The CQDs are spherical in shape with a narrow size distribution ranging from 2.0 to 4.5 nm and with an average size of 3.1 nm shown in Fig. 2b, c. Figure 2d shows the lattice spacing of 0.215 nm which corresponds to the [100] facet of graphitic carbon, and the corresponding fast Fourier transform (FFT) pattern of the CQDs further shows the highly crystalline structure, consistent with the previous report [25]. Compared with the previous studies [19, 21–23], as shown in Fig. 2, the CQDs obtained in our work not only have good quality but also show better uniform morphology. Therefore, CQDs with uniform rounded morphology and well-crystalline can be fabricated through a facile hydrothermal treatment process. The production yield (PY) of CQDs can be calculated according to the definition PY = (*m*/*M*) × 100%, where *m* is the mass of the CQDs, and *M* is the mass of fresh lemon juice. The production yield of CQDs prepared in this study is about 0.1% according to the measurement results, namely, 100 g liquid with 6.30% citric acid can obtain about 0.1 g CQDs (see Table 1).

**Fig. 3:**
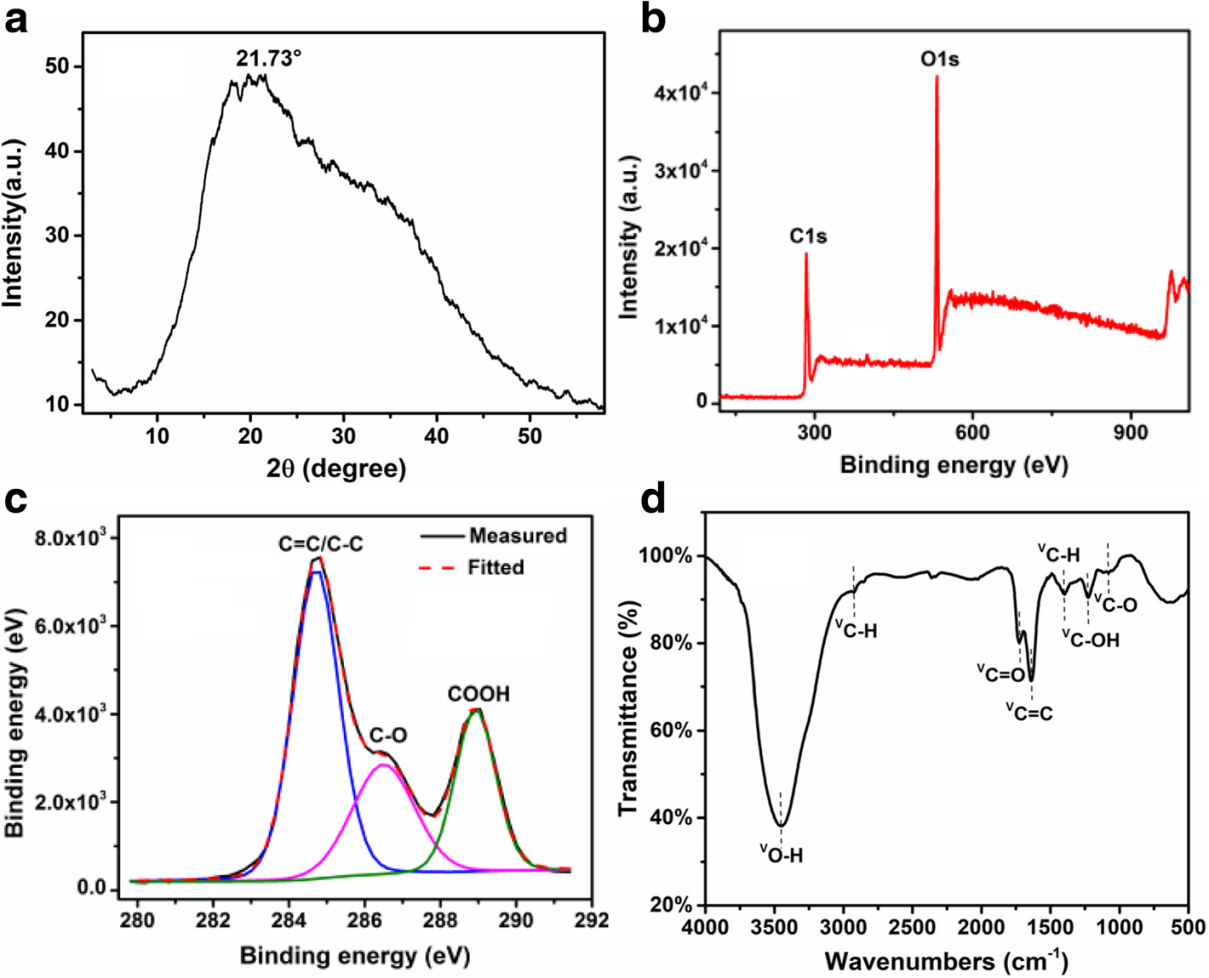
**a** XRD pattern. **b** Low-range XPS spectra. **c** XPS high-resolution scan of the C1s region. **d** FTIR spectra of CQDs

The typical X-ray diffraction (XRD) and XPS profile of CQDs are shown in Fig. 3. There is a broad (002) peak centered at 2*θ*~21.73°, and the interlayer spacing was calculated to be 0.409 nm, corresponding to the graphite structure, as shown in Fig. 3a, which is similar to the reported devalues for CQDs prepared by other methods [15, 26]. The variation of interlayer distance may be caused by the introduction of more oxygen-containing groups such as the presence of –OH and –COOH on the CQD surface and edge during the procedure of hydrothermal reaction for the preparation of CQDs. XPS and FTIR were employed to detect the composition of CQDs. As shown in Fig. 3b, c, the XPS spectrum shows a dominant graphitic C1s peak at 284.5 eV and O1s peak at 531.4 eV of CQDs. The typical peak at 284.7, 286.5, and 288.9 eV in a high-resolution scan of the C1s XPS spectrum (Fig. 3c is attributed to the C=C/C–C, C–O and C=O/COOH, respectively. It clearly indicates that CQDs were functionalized with hydroxyl, carbonyl, and carboxylic acid groups, which are beneficial to the surface modification and functionalization, and is also conducive to the solubility in water. Figure 3d shows the Fourier transform infrared spectroscopy (FTIR) spectrum of the CQDs. The presence of oxygen functionalities of different types in CQDs was confirmed by peaks at 3450 cm^−1^ (O–H stretching vibrations), 2927 cm^−1^, 1407 cm^−1^ (C–H stretching vibrations), 1726 cm^−1^(C=O stretching vibrations), 1639 cm^−1^ (C=C stretching vibrations), 1227 cm^−1^ (C–OH stretching vibrations), and 1080 cm^−1^ (C–O stretching vibrations). It is noticed that the FTIR analysis is in align with the above XPS result. Most importantly, the C–O–C (epoxy) peak disappeared completely at 1290 cm^−1^. These results imply the formation mechanism of CQDs, with the epoxy groups rupturing and the underlying C–C bonds formed, subsequently the sp^2^ domains was extracted from small molecule precursors such as glucose, fructose, ascorbic acid, and citric acid by further dehydration or carbonization and ultimately to form CQDs. Therefore, the bond scission of the surrounding oxygen groups contributes to the formation of the CQDs [15, 27].

At present, the possible mechanisms for the formation of CQDs from carbon precursors by the hydrothermal method have been proposed and examined [28]. On the base of these published results, we can understand the synthesis mechanism of CQDs from lemon juice. The pulp-free lemon juice is heated and dehydrated to form the basic framework of C=C/C–C which is mainly composed of CQDs, and the rest of the molecules reach the surface of the nucleus to produce a new C=C/C–C bond and then grown continuously in this form. With the extension of the heating time, the morphology of CQDs is gradually formed. At the same time, in the process of hydrothermal treatment to formed CQDs, the surface and edge of CQDs may contain a lot of hydroxyl (–OH), carboxyl (–COOH), and carbonyl (–C=O) or other oxygen-containing functional groups; a portion of the H and O atom in these groups could be removed by dehydrating in the hydrothermal environment.

**Fig. 4:**
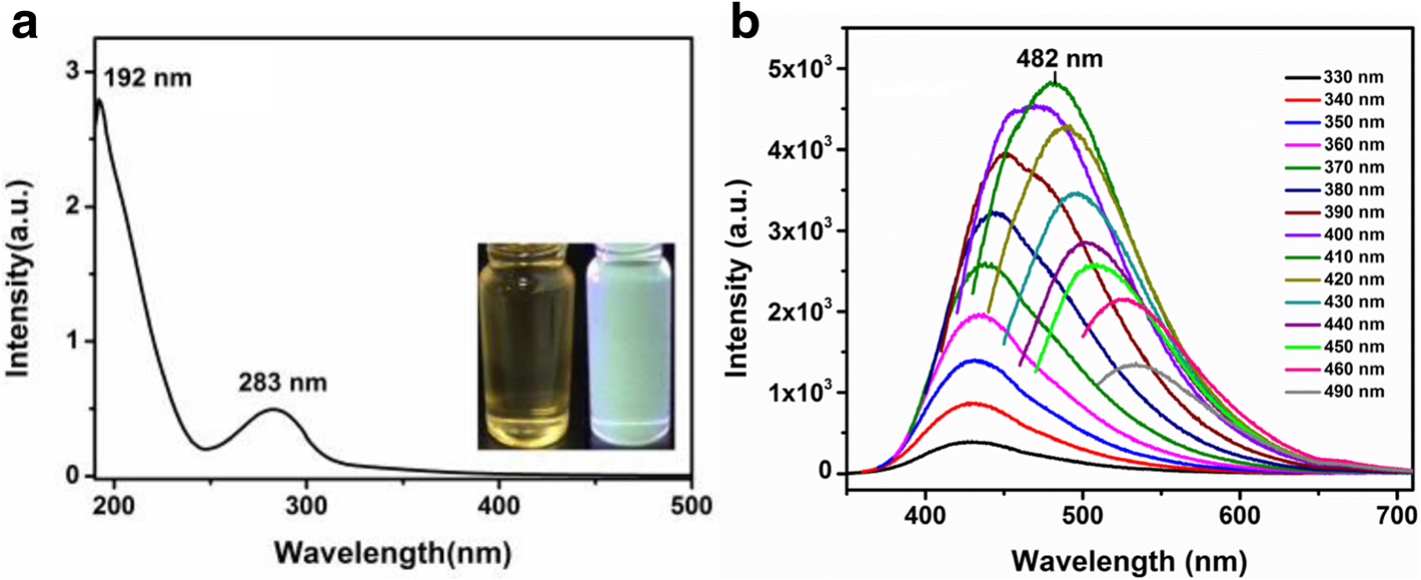
**a** UV-Vis absorption spectra of CQDs, inset: optical images under daylight (left) and UV light (right). **b** PL spectra of CQDs at different excitation wavelengths

To examine the optical properties of CQDs, ultraviolet-visible (UV-Vis) absorption and photoluminescence (PL) spectra of CQDs were measured accordingly. As shown in Fig. 4a, the optical absorption peak of the CQDs was observed in the ultraviolet region with a maximum absorption at 283 nm, which is due to *n*-π* transition of the C=O band [29]. The PL spectrum in Fig. 4b shows that the PL emission wavelength of CQDs reaches the peak at 482 nm with an excitation wavelength of 410 nm. The emission wavelength shifted from 430 to 530 nm when the excitation wavelength was increased from 330 to 490 nm. With the increase of excitation wavelength, fluorescence emitting peaks turn to redshift, referring to the occurrence of photon reabsorption. The result reveals that CQDs has an excitation-dependent PL feature [30]. The green fluorescent CQDs also show a broad PL peak that shifts with the change of excitation wavelength, which is related to the quantum-confinement effect and edge defects. With the standard PL measurement [22], the fluorescence quantum yield of the CQDs is 16.7% with an excitation wavelength of 410 nm, where quinine sulfate had been used as the reference. This value is significantly better than the QY (8.95%) of CQDs made from lemon juice in the previous report [24]. It is known that the QY of the CQDs can be dramatically enhanced after surface modification or passivation [30]. The adding of ethanol during the synthesization process can introduce more functional groups which can result in a higher QY of CQDs. However, the QY of the CQDs in this study is markedly lower than the QY of the CQDs synthesized by using citric acid (CA) and ethanolamine (EA) as the model molecules. Here, pyrolysis at 180 °C resulted in a molecular precursor with a strongly intense PL and high QY of 50%, which is due to the N doping during the synthesis process [30].

**Fig. 5:**
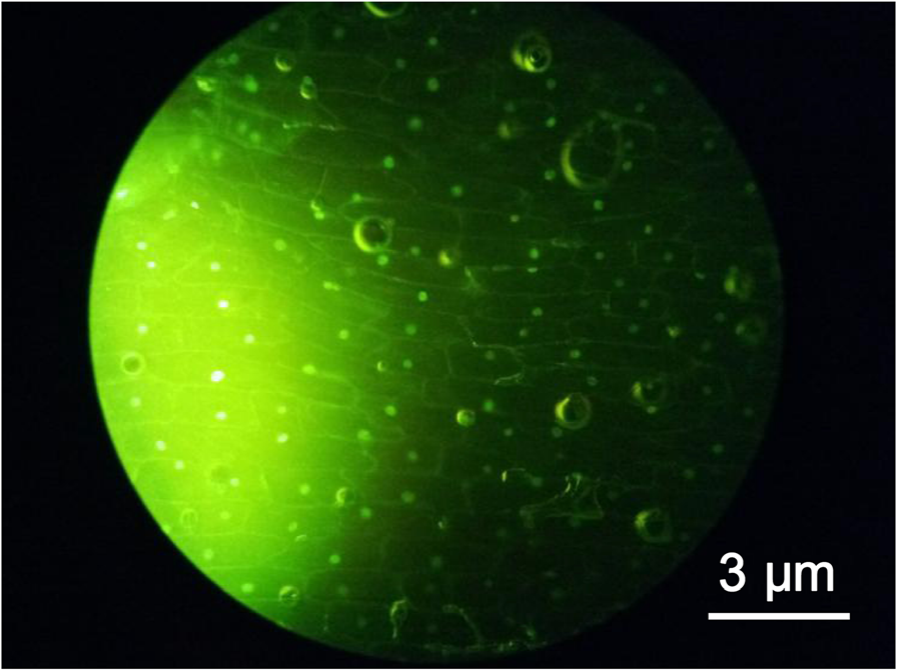
An optic image of onion epidermal cells dyed using CQDs illuminated by a blue light source

Being non-toxic and environment-friendly, the CQDs are considered as alternatives for semiconductor quantum dots to be applied in biological systems both in vitro and in vivo. The as-synthesized CQDs were applied in an optical image of onion epidermal cells as shown in Fig. 5. The fluorescence microscopy reveals that the cell walls and cell nucleus of the inner epidermal cells of the onion can be seen clearly, well-bedded and strong in three-dimensional sense. The results show that the staining and imaging of carbon quantum dots are excellent and have no adverse effect on organisms and no morphological damages of the cells observed, further demonstrating CQDs with low cytotoxicity. The confocal image in Fig. 5 indicates that the CQDs synthesized from lemon juice can be used in the plant cell imaging as fluorescent indicators, moreover showing the potential applications of CQDs biological imaging.

## Conclusions

In this study, the water-soluble fluorescent carbon quantum dots have been synthesized using lemon juice as carbon resource by a facile hydrothermal reaction. These CQDs are with good material and optical properties. They can emit bright blue-green color fluorescence under UV or blue light irradiation. We have demonstrated that the CQDs can be used in imaging of plant cells. We hope these important and significant findings can help us to gain an in-depth understanding of CQDs and to explore more practical applications of the newly carbon-based nanostructures.

## Abbreviations

CQDs: Carbon quantum dots
FFT: Fast Fourier transform
HPLC: High-performance liquid chromatography
PL: Photoluminescence
QLED: Quantum dot LED
QY: Quantum yield
R-CDs: Red-emitting carbon dots
TEM: Transmission electron microscope
UV-Vis: Ultraviolet-visible
XPS: X-ray photoelectron spectroscopy
XRD: X-ray diffraction
